# 2-Bromo-3-hy­droxy-6-methyl­pyridine

**DOI:** 10.1107/S1600536813029498

**Published:** 2013-11-06

**Authors:** Govind Pratap Singh, N. Rajesh Goud, P. Jeevan Kumar, C. N. Sundaresan, G. Nageswara Rao

**Affiliations:** a Department of Chemistry, Sri Sathya Sai Institute of Higher Learning, Prasanthinilayam, Ananthapur, Andhra Pradesh, 515 134, India; b School of Chemistry, University of Hyderabad, Gachibowli, Hyderabad, 500 046, India; c Department of Chemistry, Sri Sathya Sai Institute of Higher Learning, Brindavan Campus, Whitefield, Bangalore, India

## Abstract

In the title compound, C_6_H_6_BrNO, the Br atom is displaced from the pyridine ring mean plane by 0.0948 (3) Å, while the hydroxyl O atom and the methyl C atom are displaced by 0.0173 (19) and 0.015 (3) Å, respectively. In the crystal, mol­ecules are linked *via* O—H⋯N hydrogen bonds, forming chains propagating along the *a*-axis direction. These chains are linked by C—H⋯Br hydrogen bonds, forming corrugated two-dimensional networks lying parallel to the *ac* plane.

## Related literature

3-Hy­droxy­pyridine, the core skeleton of the title compound is an integral part of Nikkomycin Z (a potent fungicide), see: Tetsu *et al.* (1990 ▶). For the synthesis, see: Kjell *et al.* (1969 ▶).
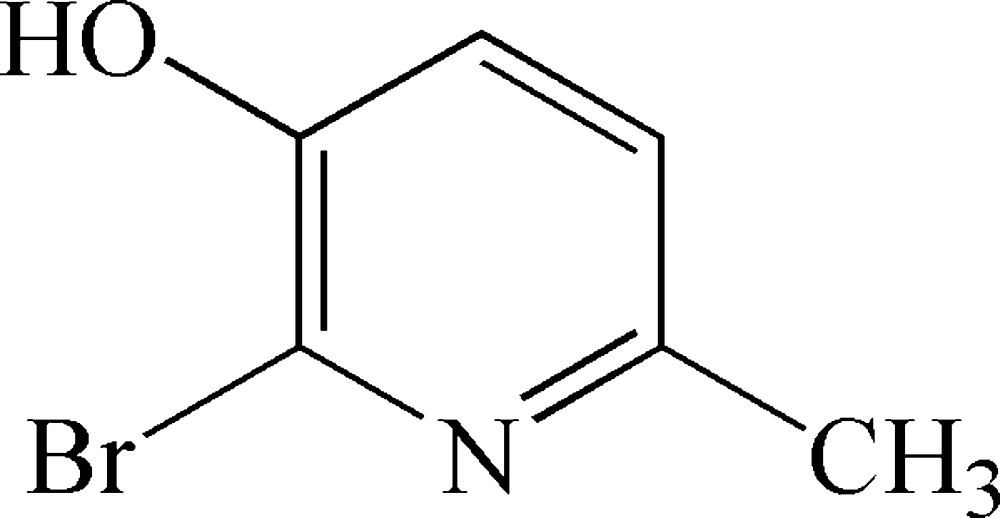


## Experimental

### 

#### Crystal data

C_6_H_6_BrNO
*M* *_r_* = 188.03
Orthorhombic, 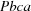

*a* = 11.4484 (19) Å
*b* = 9.0914 (15) Å
*c* = 13.230 (2) Å
*V* = 1377.1 (4) Å^3^
*Z* = 8
Mo *K*α radiation
μ = 5.88 mm^−1^
*T* = 298 K
0.32 × 0.22 × 0.12 mm

#### Data collection

Bruker SMART CCD area-detector diffractometer
Absorption correction: multi-scan (*SADABS*; Bruker, 2001 ▶) *T*_min_ = 0.255, *T*_max_ = 0.539
12822 measured reflections
1335 independent reflections
1115 reflections with *I* > 2σ(*I*)
*R*_int_ = 0.032

#### Refinement

*R*[*F* ^2^ > 2σ(*F* ^2^)] = 0.025
*wR*(*F* ^2^) = 0.066
*S* = 1.06
1335 reflections
87 parameters
H atoms treated by a mixture of independent and constrained refinement
Δρ_max_ = 0.22 e Å^−3^
Δρ_min_ = −0.36 e Å^−3^

### 

Data collection: *SMART* (Bruker, 2001 ▶); cell refinement: *SAINT* (Bruker, 2001 ▶); data reduction: *SAINT*; program(s) used to solve structure: *SHELXS97* (Sheldrick, 2008 ▶); program(s) used to refine structure: *SHELXL97* (Sheldrick, 2008 ▶); molecular graphics: *SHELXTL* (Sheldrick, 2008 ▶); software used to prepare material for publication: *SHELXTL* and *X-SEED* (Barbour, 2001 ▶).

## Supplementary Material

Crystal structure: contains datablock(s) I. DOI: 10.1107/S1600536813029498/su2659sup1.cif

Structure factors: contains datablock(s) I. DOI: 10.1107/S1600536813029498/su2659Isup2.hkl

Supplementary material file. DOI: 10.1107/S1600536813029498/su2659Isup3.cdx

Supplementary material file. DOI: 10.1107/S1600536813029498/su2659Isup4.cml

Additional supplementary materials:  crystallographic information; 3D view; checkCIF report

## Figures and Tables

**Table 1:** Hydrogen-bond geometry (Å, °)

*D*—H⋯*A*	*D*—H	H⋯*A*	*D*⋯*A*	*D*—H⋯*A*
O1—H1⋯N1^i^	0.80 (3)	1.92 (3)	2.717 (3)	174 (3)
C6—H6*B*⋯Br1^ii^	0.96	3.04	3.993 (3)	174

Symmetry codes: (i) 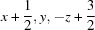; (ii) 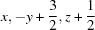.

**Fig. 1.**
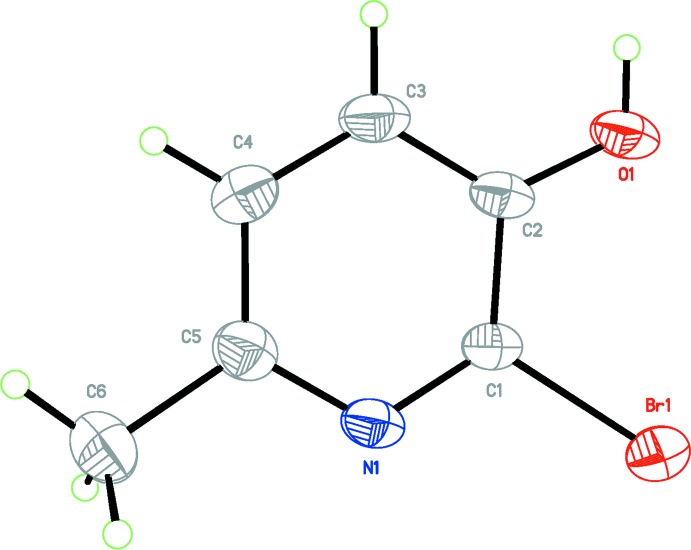
A view of the molecular structure of the title molecule, with atom labelling. Displacement ellipsoids are drawn at the 35% probability level.

**Fig. 2.**
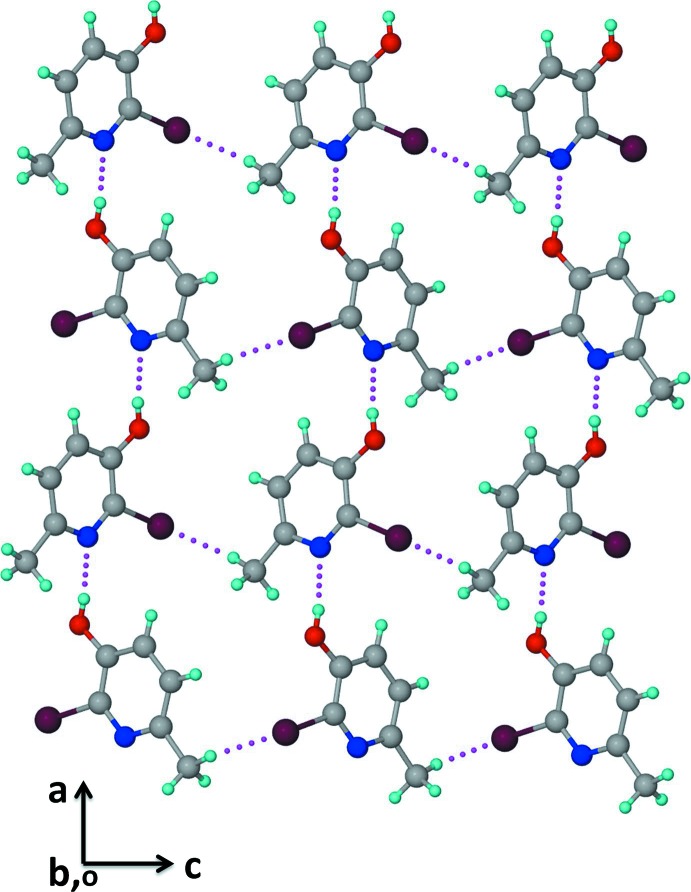
A view normal to the ac plane of the crystal packing of the title compound. Hydrogen bonds are shown as dashed lines (see Table 1 for details; a axis vertical; c axis horizontal).

## supplementary crystallographic information

### 1. Comment

3-Hydroxypyridine is an integral part of Nikkomycin *Z* (NZ), a potent fungicide, insecticide, miticide, and inhibitor of fungal and insect chitin synthetase (Tetsu *et al.*, 1990). Various biaryl derivative compounds, derived originally from 3-hydroxypyridine, are PDE4 inhibitors useful for the treatment and prevention of strokes, myocardial infarction and cardiovascular inflammatory diseases and disorders. Herein we describe the crystal structure of the 2-bromo derivative of 3-hydroxy-6-methylpyridine, previously synthesized by (Kjell *et al.*, 1969).

The molecular structure of the title molecule is illustrated in Fig. 1. The bond lengths and angles are normal.

In the crystal, molecules are linked via O-H···N hydrogen bonds forming chains propagating along the a axis direction (Fig. 2 and Table 1). These chains are linked by weak C-H···Br hydrogen bonds forming corrugated two-dimensional networks lying parallel to the ac plane (Fig. 2 and Table 1).

### 2. Experimental

The title compound was synthesized following the published procedure (Kjell *et al.*, 1969). Colourless crystals suitable for X-ray diffraction analysis were obtained by slow evaporation of a solution of the title compound in ethanol [m.p. = 460–462 K].

### Crystal data

**Table:**

C_6_H_6_BrNO	*F*(000) = 736
*M**_r_* = 188.03	*D*_x_ = 1.814 Mg m^−^^3^
Orthorhombic, *P**b**c**a*	Mo *K*α radiation, λ = 0.71073 Å
Hall symbol: -P 2ac 2ab	Cell parameters from 4373 reflections
*a* = 11.4484 (19) Å	θ = 3.1–25.7°
*b* = 9.0914 (15) Å	µ = 5.88 mm^−^^1^
*c* = 13.230 (2) Å	*T* = 298 K
*V* = 1377.1 (4) Å^3^	Needle, colorless
*Z* = 8	0.32 × 0.22 × 0.12 mm

### Data collection

**Table:**

Bruker SMART CCD area-detector diffractometer	1335 independent reflections
Radiation source: fine-focus sealed tube	1115 reflections with *I* > 2σ(*I*)
Graphite monochromator	*R*_int_ = 0.032
phi and ω scans	θ_max_ = 25.9°, θ_min_ = 3.1°
Absorption correction: multi-scan (*SADABS*; Bruker, 2001)	*h* = −14→14
*T*_min_ = 0.255, *T*_max_ = 0.539	*k* = −11→11
12822 measured reflections	*l* = −16→16

### Refinement

**Table:**

Refinement on *F*^2^	Primary atom site location: structure-invariant direct methods
Least-squares matrix: full	Secondary atom site location: difference Fourier map
*R*[*F*^2^ > 2σ(*F*^2^)] = 0.025	Hydrogen site location: inferred from neighbouring sites
*wR*(*F*^2^) = 0.066	H atoms treated by a mixture of independent and constrained refinement
*S* = 1.06	*w* = 1/[σ^2^(*F*_o_^2^) + (0.0376*P*)^2^ + 0.3754*P*] where *P* = (*F*_o_^2^ + 2*F*_c_^2^)/3
1335 reflections	(Δ/σ)_max_ = 0.001
87 parameters	Δρ_max_ = 0.22 e Å^−^^3^
0 restraints	Δρ_min_ = −0.36 e Å^−^^3^

### Special details

**Table:**

Geometry. Bond distances, angles etc. have been calculated using the rounded fractional coordinates. All su's are estimated from the variances of the (full) variance-covariance matrix. The cell esds are taken into account in the estimation of distances, angles and torsion angles
Refinement. Refinement of *F*^2^ against ALL reflections. The weighted *R*-factor *wR* and goodness of fit *S* are based on *F*^2^, conventional *R*-factors *R* are based on *F*, with *F* set to zero for negative *F*^2^. The threshold expression of *F*^2^ > σ(*F*^2^) is used only for calculating *R*-factors(gt) *etc*. and is not relevant to the choice of reflections for refinement. *R*-factors based on *F*^2^ are statistically about twice as large as those based on *F*, and *R*- factors based on ALL data will be even larger.

### Fractional atomic coordinates and isotropic or equivalent isotropic displacement parameters (Å^2^)

**Table:**

	*x*	*y*	*z*	*U*_iso_*/*U*_eq_	
Br1	0.71386 (2)	1.06988 (3)	0.63569 (2)	0.0542 (1)	
O1	0.95756 (15)	0.9896 (2)	0.69349 (15)	0.0603 (7)	
N1	0.68110 (16)	0.8898 (2)	0.79970 (15)	0.0419 (6)	
C1	0.76362 (18)	0.9477 (2)	0.74339 (17)	0.0380 (6)	
C2	0.88204 (18)	0.9217 (2)	0.75492 (19)	0.0422 (7)	
C3	0.9120 (2)	0.8254 (3)	0.83193 (19)	0.0498 (8)	
C4	0.8269 (2)	0.7633 (3)	0.89095 (18)	0.0506 (8)	
C5	0.7110 (2)	0.7963 (3)	0.87433 (17)	0.0466 (8)	
C6	0.6136 (2)	0.7345 (4)	0.9362 (2)	0.0664 (10)	
H1	1.023 (3)	0.961 (3)	0.700 (2)	0.075 (10)*	
H3	0.99010	0.80290	0.84360	0.0600*	
H4	0.84740	0.69860	0.94240	0.0610*	
H6A	0.57840	0.81170	0.97520	0.1000*	
H6B	0.64370	0.66040	0.98090	0.1000*	
H6C	0.55610	0.69160	0.89240	0.1000*	

### Atomic displacement parameters (Å^2^)

**Table:**

	*U*^11^	*U*^22^	*U*^33^	*U*^12^	*U*^13^	*U*^23^
Br1	0.0429 (2)	0.0644 (2)	0.0552 (2)	0.0084 (1)	−0.0049 (1)	0.0091 (1)
O1	0.0266 (9)	0.0798 (13)	0.0746 (13)	0.0023 (8)	0.0044 (8)	0.0172 (11)
N1	0.0281 (8)	0.0498 (10)	0.0479 (11)	−0.0018 (8)	−0.0013 (8)	−0.0035 (9)
C1	0.0286 (10)	0.0420 (12)	0.0434 (11)	0.0032 (9)	−0.0032 (9)	−0.0038 (9)
C2	0.0252 (10)	0.0493 (13)	0.0520 (13)	−0.0004 (9)	−0.0013 (9)	−0.0039 (10)
C3	0.0304 (11)	0.0610 (15)	0.0580 (14)	0.0062 (11)	−0.0076 (10)	−0.0011 (12)
C4	0.0447 (13)	0.0576 (15)	0.0496 (13)	0.0047 (12)	−0.0095 (10)	0.0038 (12)
C5	0.0395 (13)	0.0516 (14)	0.0486 (14)	−0.0036 (10)	0.0007 (10)	−0.0032 (10)
C6	0.0535 (15)	0.0822 (19)	0.0634 (17)	−0.0139 (14)	0.0056 (13)	0.0121 (15)

### Geometric parameters (Å, º)

**Table:**

Br1—C1	1.894 (2)	C4—C5	1.378 (3)
O1—C2	1.338 (3)	C5—C6	1.493 (4)
O1—H1	0.80 (3)	C3—H3	0.9300
N1—C5	1.347 (3)	C4—H4	0.9300
N1—C1	1.313 (3)	C6—H6A	0.9600
C1—C2	1.385 (3)	C6—H6B	0.9600
C2—C3	1.386 (3)	C6—H6C	0.9600
C3—C4	1.370 (3)		
			
C2—O1—H1	113 (2)	N1—C5—C4	119.9 (2)
C1—N1—C5	119.07 (19)	C2—C3—H3	120.00
Br1—C1—N1	116.45 (15)	C4—C3—H3	120.00
N1—C1—C2	125.0 (2)	C3—C4—H4	120.00
Br1—C1—C2	118.52 (16)	C5—C4—H4	120.00
O1—C2—C1	119.2 (2)	C5—C6—H6A	109.00
C1—C2—C3	115.5 (2)	C5—C6—H6B	109.00
O1—C2—C3	125.3 (2)	C5—C6—H6C	110.00
C2—C3—C4	120.2 (2)	H6A—C6—H6B	109.00
C3—C4—C5	120.3 (2)	H6A—C6—H6C	109.00
N1—C5—C6	116.7 (2)	H6B—C6—H6C	109.00
C4—C5—C6	123.4 (2)		
			
C5—N1—C1—Br1	176.80 (17)	N1—C1—C2—O1	−178.9 (2)
C5—N1—C1—C2	−0.8 (3)	C1—C2—C3—C4	−0.2 (3)
C1—N1—C5—C6	179.7 (2)	O1—C2—C3—C4	179.3 (2)
C1—N1—C5—C4	0.3 (3)	C2—C3—C4—C5	−0.1 (4)
Br1—C1—C2—C3	−176.81 (17)	C3—C4—C5—C6	−179.2 (3)
Br1—C1—C2—O1	3.6 (3)	C3—C4—C5—N1	0.1 (4)
N1—C1—C2—C3	0.7 (3)		

### Hydrogen-bond geometry (Å, º)

**Table:**

*D*—H···*A*	*D*—H	H···*A*	*D*···*A*	*D*—H···*A*
O1—H1···N1^i^	0.80 (3)	1.92 (3)	2.717 (3)	174 (3)
C6—H6*B*···Br1^ii^	0.96	3.04	3.993 (3)	174

Symmetry codes: (i) *x*+1/2, *y*, −*z*+3/2; (ii) *x*, −*y*+3/2, *z*+1/2.
